# Monitoring of ticks and their pathogens from companion animals obtained by the “tekenscanner” application in The Netherlands

**DOI:** 10.1007/s00436-022-07518-3

**Published:** 2022-04-22

**Authors:** F. N. J. Kooyman, H. Zweerus, E. R. Nijsse, F. Jongejan, J. A. Wagenaar, E. M. Broens

**Affiliations:** 1 Department Biomolecular Health Sciences, Faculty of Veterinary Medicine, Utrecht University, Utrecht, The Netherlands; 2 Department of Veterinary Tropical Diseases, Faculty of Veterinary Sciences, University of Pretoria, Private Bag X40, Onderstepoort, 0110 South Africa

**Keywords:** Cats, Citizen science, Dogs, Smartphone app, Ticks, Tick-borne pathogens

## Abstract

Ticks are vectors for many pathogens of veterinary and medical interest. In order to monitor ticks and tick-borne pathogens, the “Tekenscanner” (Dutch for Tick scanner), a citizen science project, was launched in The Netherlands. It is a smartphone application for pet-owners to get ticks from their dog or cat, identified and checked for pathogens for free. At the same time, information about the pet and the geographic location of tick infestation becomes available for research. The application was launched in 2018, and the results of the first 6 months after launch of the app were reported. Ticks were identified based on morphology, and DNA was extracted and amplified by a panel of tick-borne pathogen-specific primers. Next, the amplicons were subjected to reverse line blot with specific probes for important pathogens to determine their presence or absence. The present paper describes the results of 2019 and 2020. There were 2260 ticks collected from 871 dogs and 255 cats (26 ticks were from an unknown host) and all pet owners were informed about the results. Four species of ticks were collected: *Ixodes ricinus* (90.0%), *Ixodes hexagonus* (7.3%), *Dermacentor reticulatus* (2.8%) and *Rhipicephalus sanguineus* (0.1%). *Ixodes ricinus* was the tick with the most divergent pathogens: *Anaplasma* sp. (1.3%), *Babesia* sp. (0.8%), *Borrelia* spp. (4.8%), *Neoehrlichia* sp. (3.7%) and *Rickettsia helvetica* (12.6%). In *I. hexagonus, R. helvetica* (1.8%) and *Babesia* sp. (0.6%) were detected and *Rickettsia raoultii* in *D. reticulatus* (16.2%). One of the two nymphs of *R. sanguineus* was co-infected with *Borrelia* and *R. helvetica* and the other one was uninfected. The high numbers of different pathogens found in this study suggest that companion animals, by definition synanthropic animals, and their ticks can serve as sentinels for emerging tick-borne pathogens.

**Supplementary Information:**

The online version contains supplementary material available at 10.1007/s00436-022-07518-3.

## Introduction

Ticks are the important vectors for several pathogens of veterinary and medical interest. The role of ticks as vectors for *Babesia*, *Borrelia* and *Anaplasma* species is well described (Beugnet and Marié 2009), and many of these pathogens can be found in companion animals like cats and dogs with possible implications for general public health (Skotarczak 2018). Ticks from these animals can serve as sentinels for vectors and pathogens of veterinary importance, and because their synanthropic lifestyle, for medical relevance. In the UK, a large survey among 1278 veterinary practices resulted in the collection of 7106 ticks retrieved from cats and dogs (Abdullah et al. 2016; Davies et al. 2017). The dominant tick species from dogs was *Ixodes ricinus*. The ticks obtained from the cats were screened for the presence of TBPs and DNA of several *Borrelia* and *Babesia* species was detected.

Recently, a very large survey on ticks collected by pet owners was conducted by citizen science approach in the USA. More than 16,000 ticks collected by pet-owners from 49 states (all except Alaska) were analysed (Nieto et al. 2018). Through this citizen project, extensive sampling was facilitated and the results provided a good view of the geographical distribution of the tick species and TBPs. However, this approach has limitations as well. The mere presence of pathogen DNA in a feeding tick is no evidence that it has acquired the pathogen from the host of which it was removed, nor that the tick can transmit or has transmitted the pathogen to that host. The prevalence of ticks over the pet population cannot be determined, because the proportion of pets without ticks is not known, just as the absence/presence of the TBP within the host. Despite these disadvantages, this manner of acquisition of ticks from cats and dogs turned out to be a cost-effective way to monitor ticks and TBPs (Estrada-Peña et al. 2021), especially considering the high social cost related to the traditional tick sampling systems (Capelli et al. 2012).

To involve pet-owners in the collection of ticks from their pets, the “Tekenscanner” (Dutch for Tick scanner) smartphone application, funded by Bayer was launched in 2018 in the Netherlands (Jongejan et al. 2019). Pet-owners collected ticks from their dogs or cats and sent them, together with information of place and date of collection and pet species, age and race to the laboratory for free identification of the ticks and the pathogens. In 2018, 1050 ticks were collected from cats and dogs of which the majority was *I. ricinus* (90.0%). *Rickettsia helvetica* DNA (8.4%) was detected most frequently in *I. ricinus* from both cats and dogs, next to DNA of several *Babesia* and *Borrelia* species. In *Dermacentor reticulatus* the pathogen *Rickettsia raoultii* (13.3%) was found most often.

Epidemiology of ticks and TBPs are changing rapidly due to the climate change and change of land use and globalisation. Therefore, continuous monitoring of ticks and TBP can be used to follow trends over time. The present study describes this monitoring of ticks and TBP in The Netherlands by application of the “Tekenscanner” from 2019 and 2020 using a slightly modified method.

## Materials and method

### Ticks and pathogens

Collection, submission, identification and processing of ticks were performed as described by Jongejan et al. (2019). Ticks were collected mostly in the Netherlands and a small number of ticks were from other European countries. In short, pet owners and practitioners used the “Tekenscanner” app to register one or more ticks that were removed from their dog or cat, data on age, sex and breed of their pet was entered and ticks were submitted to the laboratory. Ticks were identified using a binocular microscope with 80 × magnification and processed further for pathogen detection. In contrary to Jongejan et al. (2019), ticks from the same host were not pooled but processed individually.

### PCR and reverse line blot (RLB)

PCR and Reverse Line Blot (RLB) were performed as described using the same primers and probes (Jongejan et al. 2019) with some modifications. In 2020, the *Borrelia* reverse primer was replaced by new primer 5’-biotin-GAG AGT AGG TTA TTG CCA GGG-3’ (Rijpkema et al. 1995), as an unintended insertion (5′-biotin-GAG AGT AGG TTA TTG GCCA GGG-3′) was noticed in the primer that was used in 2018 and 2019. All PCRs were performed using Phusion U green multiplex PCR master mix (ThermoScientific) with the following conditions for all reactions: after an initial denaturation at 98 °C for 30 s, 10 cycles of 98 °C (10 s), 64 to 55 °C (30 s) and 72 °C (15 s) were performed, in which the annealing temperature decreased with 1 °C in each cycle. Finally, 49 cycles with annealing temperature of 54 °C were performed with a final extension of 72 °C step for 7 min. PCRs for *Babesia*/*Theileria* and *Anaplasma*/*Ehrlichia*/*Rickettsia* were performed as multiplex, whereas the *Borrelia* PCR was performed separately. Prior to the RLB hybridisation, the two PCR products from the same tick were combined and RLB was performed as described (Jongejan et al. 2019).

### Statistics

Differences in prevalence were, when appropriate, evaluated with Fisher’s exact test. A *p* value less than 0.05 was considered statistically significant.

### Sequencing and DNA analysis

When appropriate, PCR products were treated with Exo-SAP-IT (affymetrics) and sent to Baseclear (Leiden, The Netherlands) for Sanger sequencing in both directions with the same primers as used for PCR. Sequences were aligned in SeqMan Pro 14 (DNASTAR lasergene v16) and blasted (BLASTn) against the non-redundant nucleotide collection (nr/nt) at NCBI.

## Results

Over the study period, 2286 ticks were recovered from 871 dogs, 255 cats and 22 unknown hosts and DNA of 16 pathogens was detected.

### Tick species

**Fig. 1:**
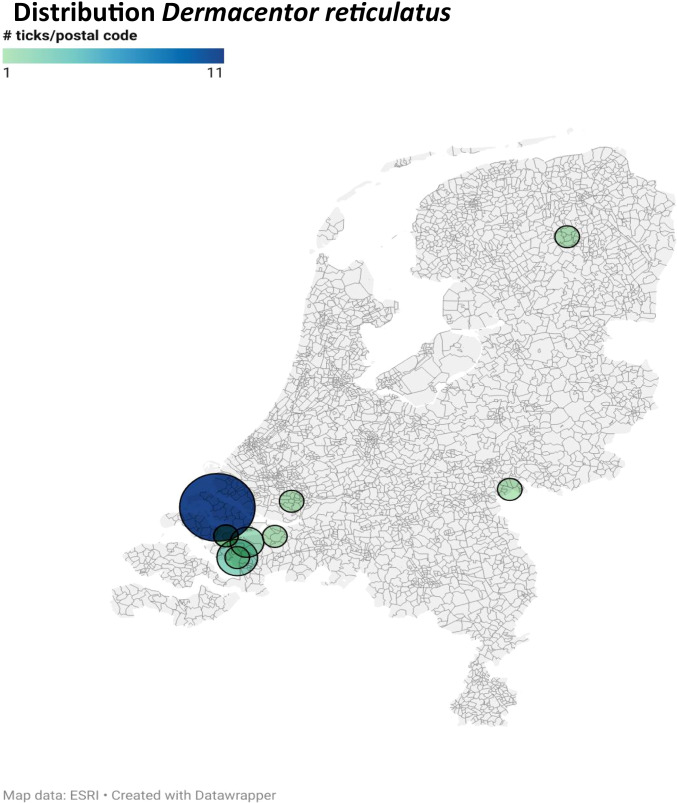
Distribution of *Dermacentor reticulatus* over the Netherlands. The large, blue circle represents 11 ticks per postal code, the smallest, light green circles, represent 1 tick per postal code. created by datawrapper (https://www.datawrapper.de)

Ticks (n = 1902) from 686 dogs (1375 ticks), 201 cats (501 ticks) and 22 unreported hosts (26 ticks) were received in 2019 (Table 1). Due to covid-19 pandemic measures, less ticks were received in 2020, only 290 ticks from 185 dogs and 94 ticks from 54 cats were collected (Table 2). More detailed data are given in Supplementary Table S1. All tick stages were found during the study period; adult females were the most abundant (82.3%), followed by the adult males (9.7%), nymphs (6.8%) and larvae (1.2%). Twenty-six of the 27 larvae were recovered from cats; only one larva came from a dog. About 2% of ticks (41/2286) were collected outside The Netherlands, Belgium (10), Luxembourg (1), Germany (23), Denmark (2), Slovenie (2) and France (3). Four species of ticks were found: *I. ricinus* (90.0%), *Ixodes hexagonus* (7.3%), *D. reticulatus* (2.8%) and *Rhipicephalus sanguineus* (0.1%) during the study period. From dogs and cats, in total respectively 1545 and 485 *I. ricinus* ticks were identified and all stages were found. There were 166 *I. hexagonus* ticks analysed. No *I. hexagonus* males were found. *Dermacentor reticulatus* ticks were almost exclusively found on dogs (57/64) and most of them came from the South-Western part of the country (Fig. 1). Two *R. sanguineus* ticks were found, both were nymphs of which one was collected in France.

**Table 1:** Number of ticks and their hosts obtained in 2019

			Tick species		
Host	*Ixodes* *ricinus*	*Ixodes* *hexagonus*	*Dermacentor* *reticulatus*	*Rhipicephalus* *sanguineus*	All tick *species*
Dogs (n = 686)	1278	48	48	1	1375
Cats (n = 201)	406	94	1	0	501
Unknown (n = 22)	24	0	2	0	26
**All (n** = **909)**	**1708**	**142**	**51**	**1**	**1902**

Numbers in bold represent the totals of the column

**Table 2:** Number of ticks and their hosts obtained in 2020

			Tick species		
	*Ixodes* *ricinus*	*Ixodes* *hexagonus*	*Dermacentor* *reticulatus*	*Rhipicephalus* *sanguineus*	All tick species
Dogs (n = 185)	*267*	13	9	1	290
Cats (n = 54)	79	11	4	0	94
**All (n** = **239)**	**346**	**24**	**13**	**1**	**384**

Numbers in bold represent the totals of the column

### Ixodus ricinus and pathogens

Pathogens were detected in 21.4% of *I. ricinus* ticks (Table 3) and in 2.4% of the *I. ricinus* ticks more than one pathogen was detected (Supplementary Table S1). *Rickettsia* spp. were most often found, with *R. helvetica* being the most abundant species with a prevalence of 11.5% in ticks from dogs and 14.4% in ticks from cats (not significantly different, p = 0.0942). *Rickettsia raoultii* was found once in an adult male from a dog and one female tick from a dog yielded a PCR product that hybridised with the catch-all probe for *Rickettsia*, but with none of the *Rickettsia* species specific probes. Sequencing and blasting of the PCR product gave 93% identity with *Rickettsia monacensis* (accession number MH618378). *Neoehrlichia mikurensis* was found in ticks from dogs and cats but was significantly (p < 0.001) more abundant in ticks from dogs (4.5%) than from cats (1.0%). *Borrelia* spp. were found in 4.7% of *I. ricinus* ticks with *Borrelia garinii*, *Borrelia valaisiana* and *Borrelia afzelii*, as the most abundant species in both cats and dogs. With the *borrelia* primer containing the unintended insertion, 5.2% of the *I. ricinus* ticks were positive for *Borrelia* spp. Using the new primer, resulted in a lower proportion *Borrelia* positive *I. ricinus* ticks (2.6%). *Anaplasma phagocytophilum* was found in 26 ticks (1.3%), see Table 3, and only 16 *I. ricinus* ticks (0.8%) contained *Babesia* DNA of which the majority was *B. divergens* (n = 11).

**Table 3:** Number of *I. ricinus *ticks from dogs and cats and tick borne pathogens detected during the study period (percentage of positive ticks)

			Dogs					Cats		
pathogens	larva	nymph	male	female	all stages	larva	nymph	male	female	all stages
	n=0	n=26	n=151	n=1368	n=1545	n=11	n=22	n=49	n=403	n=485
*Anaplasma phagocytophilum*			3 (2.0)	19 (1.4)	22 (1.4)				4 (1.0)	4 (0.8)
*Anaplasma platys*			1 (0.7)		1 (0.1)					
*Neoehrlichia mikurensis*		1(3.8)	8 (5.3)	61 (4.5)	70 (4.5)				5 (1.2)	5 (1.0)
*Babesia divergens*			1 (0.7)	9 (0.7)	10 (0.6)					
*Babesia microti*				4 (0.3)	4 (0.3)				1 (0.2)	1 (0.2)
*Babesia venatorum*				1 (0.1)	1 (0.1)					
*Borrelia burgdorferi *(s.s.)		1 (3.9)	1 (0.7)	5 (0.4)	7 (0.5)		1 (4.5)		2 (0.5)	3 (0.6)
*Borrelia afzelii*			5 (3.3)	17 (1.2)	22 (1.4)			1 (2.0)	5 (1.2)	6 (1.2)
*Borrelia bissetti/carolinensis*			2 (1.3)	3 (0.2)	5 (0.3)					
*Borrelia garinii*			5 (3.3)	23 (1.7)	28 (1.8)		1 (4.5)		8 (2.0)	9 (1.9)
*Borrelia spielmanii*			5 (3.3)	8 (0.6)	13 (0.8)				3 (0.7)	3 (0.6)
*Borrelia valaisiana*			4 (2.6)	23 (1.7)	27 (1.7)			3 (6.1)	3 (0.7)	6 (1.2)
other *Borrelia burgdorferi *(s.l.)				1 (0.1)	1 (0.1)					
*Rickettsia helvetica*		3 (11.5)	17 (11.2)	157 (11.5)	177 (11.5)	1 (9.1)	2 (9.1)	5 (10.2)	62 (15.4)	70 (14.4)
Rickettsia monacensis *				1(0.1)	1 (0.1)					
*Rickettsia raoultii*			1 (0.7)		1 (0.1)					

^*^ no specific RLB probe present, but identification based on hybridization with *Rickettsia *catch-all probe and on the sequence of PCR product

### Other tick species and pathogens

In only four *I. hexagonus* ticks (2.4%, see Supplementary Table S1) pathogens were detected. Two female *I. hexagonus* ticks from a dog (1.9%) and one nymph from a cat (1.2%) contained *R. helvetica* (1.9%) and one nymph from a dog contained *Babesia canis* (4.3%).

Ten *D. reticulatus* ticks (16.1%) were infected with *R. raoultii* (Supplementary data Table S1). No other pathogens were found in these ticks.

Of the two *R. sanguineus* nymphs, only one was infected (*B. afzelii* and *R. helvetica*, see Supplementary data Table S1).

## Discussion

In 2019 and 2020, there were in total 2286 ticks collected and in 454 ticks (19.9%) pathogens were detected. Due to covid-19 measures in 2020 the number of collected ticks was much lower. *Ixodes ricinus* (90.0%) was the predominant tick species just as it was in 2018, the first year of the “tekenscanner” project (Jongejan et al. 2019). The proportion of *I. hexagonus* was for both periods, 2018 and 2019/2020 the same (7.3%) with more ticks removed from cats than from dogs. The contribution of *D. reticulatus* was also very similar, 2.4% and 2.8% for 2018 and 2019/2020, respectively, with more ticks from dogs than from cats. There were four *R. sanguineus* ticks collected in 2018 and two in 2019/2020, all removed from dogs. The four *R. sanguineus* ticks collected in 2018 were all outside The Netherlands (Italy, France, Spain and USA), and one of the two *R. sanguineus* ticks from 2019/2020 was also collected abroad (France) and did not contained pathogens. It is not known whether the dog on which the other *R. sanguineus* tick (infected with *B. afzelli* and *R. helvetica*) was found has been abroad short before collection. Analysis of the differences in the presence of pathogens in ticks of 2018 with 2019/2020 is not possible, because in 2018 ticks from the same species and host were pooled for analysis and ticks were collected only over a 6-month period.

The most frequently sent in tick species was *I. ricinus* and this tick species was more often infected with TBPs (21.4%), whereas in only 6.5% of the ticks from other species pathogen DNA could be detected.

Species from the *B. burgdorferi* s.l. complex, the causative agent of Lyme borreliosis, were exclusively found in *I. ricinus* ticks, just like in 2018 (Jongejan et al. 2019). *Borrelia burgdorferi* s.l. DNA was detected in almost 5% of the *I. ricinus* ticks. The percentage infected ticks was higher in 2019, when the primer with the unintended insertion was used, as in 2020. Therefore, the negative effect of the insertion was expected to be low. Infection with *B. burgdorferi* in dogs and cats leads to seroconversion, but direct relation with the disease development is still contentious (Littman et al. 2018). Humans are mostly bitten by *I. ricinus* nymphs (Hartemink et al. 2021), therefore the infection of *B. burgdorferi* s.l. in nymphs (4.1%) is of medical importance. We detected six species of the *B. burgdorferi* s.l. complex and the most abundant *Borrelia* species were *B. garinii*, *B. valaisiana* and *B. afzelli* in that order, *B. burgdorferi* s.s. was also detected. *Borrelia afzelli*, *B. garinii* and *Borrelia burgdorferi* s.s. were considered the most common species in dogs and man in Europe (Skotarczak 2018).

From the *Rickettsia* group, *R. helvetica* was found often and almost exclusively in *I. ricinus* ticks. *Rickettsia helvetica* may cause Mediterranean Spotted Fever-like (MSF-like) symptoms in humans and has been involved in perimyocarditis and meningitis (Portillo et al. 2015). *Rickettsia raoultii* was the only tick-borne pathogen found in *D. reticulatus* and this pathogen was found almost exclusively in this tick, apart from one infected *I. ricinus* male. *Rickettsia raoultii* belongs to the Spotted Fever Group (SFG) rickettsiae and can cause human tick-borne lymphadenopathy (TIBOLA) (Portillo et al. 2015). Not much is known about the pathogenicity of *Rickettsia* spp. in dogs and cats.

One tick yielded a PCR product that scored the highest identity with a strain of *R. monacensis.* It is not unlikely that it is indeed this species, because *R. monacensis* is also found in *I. ricinus* ticks in France and Germany (Akl et al. 2019; Simser et al. 2002). *Rickettsia monacensis* can cause MSF-like symptoms in humans (Portillo et al. 2015) and is therefore of medical importance.

*Anaplasma phagocytophilum* was found only in about 1% of the *I. ricinus* ticks. Although *A. phagocytophilum* seems to be endemic in the Netherlands (Jahfari et al. 2014), canine anaplasmosis in The Netherlands is a rare disease. This could be due to the variation in clinical symptoms and the need for sequential sampling for a definite diagnosis as shown in a confirmed case with subclinical and clinical anaplasmosis in a pack of dogs (Hovius et al. 2018). Furthermore, *A. phagocytophilum* can cause problems in a range of hosts, including humans.

*Neoehrlichia mikurensis* is an emerging pathogen. In 2010 the first human case was described (Welinder-Olsson et al. 2010). *Neoehrlichia mikurensis* was detected in 4.5% of the *I. ricinus* ticks obtained from dogs and in 1.0% of those from cats, which is significantly lower. Apart from one nymph, all the 75 infected ticks were adults. It was assumed that there is no transovarial transmission in ticks (Portillo et al. 2018), although recent research questioned that, because unfed questing *I. ricinus* larvae were found positive for *N. mikurensis* (Ondruš et al. 2020). There is a reservoir of *N. mikurensis* in wild rodents, wild boars, hedgehogs, dogs and many other mammals, but cats and some other mammals were always found negative (Portillo et al. 2018). This can be an explanation for the difference in presence of *N. mukurensis* between ticks from dogs and cats. Possibly, dogs act as a reservoir for *N. mikurensis*, while cats do not. The positive ticks obtained from cats must have acquired the infection in that case by obtaining the infection in an earlier stage on another host or by transovarial transmission.

*Babesia divergens*, *Babesia microti* and *Babesia venatorum* were found exclusively in *I. ricinus* and *B. divergens* was the most abundant. Surprisingly, *B. divergens* was not found in the “tekenscanner” study of 2018 (Jongejan et al. 2019), although all three *Babesia* species were found before in The Netherlands in *I. ricinus* (Nijhof et al. 2007). Especially, *B. divergens* and *B. venatorum* can cause human babesiosis in splenectomized people (Gray 2006; Herwaldt et al. 2003) and *B. divergens* can cause bovine babesiosis (Zintl et al. 2003). One nymph of *I. hexagonus* was found that contained DNA of *B. canis*, the causative agent of canine babesiosis. Although, *D. reticulatus* is the prime vector for *B. canis* (Pantchev et al. 2015), the pathogen has been found before in *I. hexagonus* (Estrada-Peña et al. 2017). All *D. reticulatus* ticks from the present study were negative for *B. canis.* From 2004 onwards, canine babesiosis has been recorded in The Netherlands in dogs that have never been abroad (Matjila et al. 2005). Also *B. canis* positive *D. reticulatus* were found in that study, but other tick species were not examined. Until 2013 *B. canis* positive *D. reticulatus* ticks were still found in restricted areas in The Netherlands (Jongejan et al. 2015). The tick *D. reticulatus* has a focal distribution pattern in The Netherlands (Jongejan et al. 2015; 2019) and the *D. reticulatus* ticks from the present study were also mostly from these restricted areas. Dog owners who walk their dog in another area than their immediate living environment will obviously obscure this pattern. There can also be a bias, because the dog owners and practitioners from that area are probably more aware of tick-borne diseases and, therefore, more eager to send in the ticks for analysis. Furthermore, 9.4% of the *Dermacentor* ticks were collected in Belgium or Luxembourg. In contrast, only 0.8% of all ticks were from those countries, suggesting higher numbers of *D. reticulatus* ticks in those countries than in The Netherlands.

## Conclusion

*Ixodes ricinus* is regarded as the most important vector for pathogens of veterinary and medical interest in The Netherlands (Sprong et al. 2018). This was confirmed in the present study where *I. ricinus* was by far the most abundant tick and had the highest percentage of TBPs, including *B. burgdorferi* s.l.*, R. helvetica* and *N. mikurensis*. Monitoring in the future of ticks and TBPs from synanthropic animals by “Tekenscanner” or similar approach can be a useful early warning system for changes in tick and TBP populations. It can help to create more awareness among dog and cat owners concerning ticks and tick-borne pathogens.

## Supplementary Information

Below is the link to the electronic supplementary material.

Supplementary file1 (XLS 234 KB)
